# Biochar reduces the efficiency of nitrification inhibitor 3,4-dimethylpyrazole phosphate (DMPP) mitigating N_2_O emissions

**DOI:** 10.1038/s41598-019-38697-2

**Published:** 2019-02-20

**Authors:** T. Fuertes-Mendizábal, X. Huérfano, I. Vega-Mas, F. Torralbo, S. Menéndez, J. A. Ippolito, C. Kammann, N. Wrage-Mönnig, M. L. Cayuela, N. Borchard, K. Spokas, J. Novak, M. B. González-Moro, C. González-Murua, J. M. Estavillo

**Affiliations:** 1 University of the Basque Country (UPV/EHU), Department of Plant Biology and Ecology, Apdo. 644, E-48080 Bilbao, Spain; 2 Department of Soil and Crop Sciences, C127 Plant Sciences Building, Colorado State University, Fort Collins, CO 80523-1170 USA; 3 Geisenheim University, Department of Applied Ecology, Von-Lade-Straße 1, 65366 Geisenheim, Germany; 4 University of Rostock, Faculty of Agricultural and Environmental Sciences, Grassland and Fodder Sciences, Justus-von-Liebig-Weg 6, 18059 Rostock, Germany; 5 Department of Soil and Water Conservation and Waste Management, CEBAS-CSIC. Campus Universitario de Espinardo, 30100 Murcia, Spain; 6 Institute of Geography, Soil Science/Soil Ecology, Ruhr-University Bochum, Universitätsstrasse 150, 44801 Bochum, Germany; 7 Plant Production, Natural Resources Institute Finland (Luke), 00790 Helsinki, Finland; 8 United States Department of Agriculture, Agriculture Research Service, Soil & Water Management Research Unit, 439 Borlaug Hall, 1991 Buford Circle, University of Minnesota, St. Paul, Minnesota 55108 USA; 9 United States Department of Agriculture, Agriculture Research Service, Coastal Plains Research Center, 2611 West Lucas Street, Florence, SC 29501 USA

## Abstract

Among strategies suggested to decrease agricultural soil N_2_O losses, the use of nitrification inhibitors such as DMPP (3,4-dimethylpyrazole phosphate) has been proposed. However, the efficiency of DMPP might be affected by soil amendments, such as biochar, which has been shown to reduce N_2_O emissions. This study evaluated the synergic effect of a woody biochar applied with DMPP on soil N_2_O emissions. A incubation study was conducted with a silt loam soil and a biochar obtained from *Pinus taeda* at 500 °C. Two biochar rates (0 and 2% (w/w)) and three different nitrogen treatments (unfertilized, fertilized and fertilized + DMPP) were assayed under two contrasting soil water content levels (40% and 80% of water filled pore space (WFPS)) over a 163 day incubation period. Results showed that DMPP reduced N_2_O emissions by reducing ammonia-oxidizing bacteria (AOB) populations and promoting the last step of denitrification (measured by the ratio *nosZI* + *nosZII*/*nirS* + *nirK* genes). Biochar mitigated N_2_O emissions only at 40% WFPS due to a reduction in AOB population. However, when DMPP was applied to the biochar amended soil, a counteracting effect was observed, since the N_2_O mitigation induced by DMPP was lower than in control soil, demonstrating that this biochar diminishes the efficiency of the DMPP both at low and high soil water contents.

## Introduction

Greenhouse gas (GHG) emissions to the atmosphere and their impact on global climate are one of the greatest environmental concerns of current times. As compared to the GHGs carbon dioxide (CO_2_) and methane (CH_4_), nitrous oxide (N_2_O) is likely the most important GHG associated with agricultural soils. Although the absolute quantity of soil N_2_O emitted is lower than CO_2_ and CH_4_, it has a global warming potential 298 times greater than that of CO_2_ over 100 years and accounts for 8% of total GHG emissions, becoming a powerful GHG that persists in the atmosphere for ~114 years^1^. Agriculture is the main source of the global anthropogenic N_2_O emissions^2^, largely due to the widespread use of synthetic nitrogen (N) fertilizers and their microbial transformations to N_2_O.

The main microbial N-transforming processes contributing to N_2_O formation are nitrification and denitrification^3^. Nitrification is an aerobic process where ammonium (NH_4_^+^) is oxidized to nitrate (NO_3_^−^) via nitrite (NO_2_^−^) by the enzymes ammonia monooxygenase (AMO) and nitrite oxidoreductase (NOR). The synthesis of ammonia monooxygenase is encoded by the *amoA* gene, present in ammonia-oxidizing bacteria (AOB) and ammonia-oxidizing archaea (AOA). During ammonia oxidation, N_2_O can be formed by chemical decomposition of hydroxylamine (NH_2_OH). The end product of nitrification, nitrate, can be reduced to nitrogen gas (N_2_) via the formation of NO_2_^−^, nitric oxide (NO) and N_2_O. Such sequential denitrification processes are carried out by nitrate reductase (encoded by the *narG* gene), nitrite reductases (encoded by the *nirS/nirK* genes), and nitrous oxide reductase (encoded by the *nosZ* genes) under anaerobic soil conditions^4^.

Under most soil conditions, nitrification and denitrification occur simultaneously, with the net N_2_O flux to the atmosphere as a result of both processes combined^5^. Whether denitrification or nitrification dominates depends on many different factors such as soil characteristics (texture, available carbon, pH, aeration, microbial activity) and environmental conditions (temperature and rainfall). However, a particular regulator of the contribution of both processes to total N cycling and soil N_2_O emissions is soil water filled pore space (WFPS)^6^. Nitrification is the preferential source of N_2_O fluxes from well-aerated soils, with WFPS <60%, while at oxygen-limited conditions with WFPS >70% denitrification dominates N-transformation in soils causing emission of N_2_O^7^.

Current emission concerns are focused on how to develop effective alternatives for reducing N_2_O emissions while increasing fertilizer N use efficiency. A proposed strategy to minimize N losses is the use of nitrification inhibitors (NIs), which delay the oxidation of NH_4_^+^ to NO_3_^−^ via nitrification. During the last decade 3,4–dimethylpyrazole phosphate (DMPP) has become a commonly used NI, showing some advantages compared to other NIs, e.g. its lower phytotoxicity and higher effectiveness at small application rates in comparison with other NIs such as dicyandiamide (DCD)^8,9^. The inhibitor acts by delaying the oxidation of NH_4_^+^ to NO_3_^−^ by inhibiting ammonia monooxygenase activity, resulting in the inhibited metabolic activity and growth of AOB; this can be measured by the copy numbers of the *amoA* gene. Although the specific mechanism remains unclear, it has been proposed that DMPP acts as a chelating compound reducing the availability of Cu, the co-factor of ammonia monooxygenase^10^. The reduction of N_2_O emissions by DMPP is therefore attributed to reduced nitrification rates and subsequent denitrification rates, due to the decrease of NO_3_^−^ availability.

Indeed, the effect of NIs as mitigators of N_2_O emissions depends on many environmental factors including soil water content. Barrena *et al*.^11^ observed different trends of DMPP behaviour depending on soil water content, since at WFPS <40% DMPP provoked a direct nitrification inhibiting effect, leading to decreased *amoA* gene abundance, while at WFPS >80% DMPP application failed to induce any change in *amoA* gene abundance due to dentrifier communities dominating N-transformation^6^. This suggested that the lower N_2_O emissions would be due to an unknown effect of DMPP on the non-target denitrifiers activity and/or population. In fact, Florio *et al*.^12^ described a short-term effect of DMPP on non-target microorganisms, and Torralbo *et al*.^13^ demonstrated that dimethylpyrazole-based NIs not only inhibited nitrification but also stimulated N_2_O reduction to molecular N (N_2_) via increased nitrous oxide reductase activity under high soil water content conditions.

Recent research has demonstrated that biochar may have the ability to mitigate N_2_O emissions from agricultural soils^14,15^. In fact, the structure of biochar is known to have several capabilities that could explain its N_2_O mitigation mechanisms. It may enhance soil aeration by improving water holding capacity, increase soil pH, favour N immobilization, interact with available organic C and N in soil, modify enzymatic activities as well as potentially induce a toxic effect on nitrifier and denitrifier communities^14,16^. Even though many different explanations have been suggested, the mechanism by which biochar affects soil N-cycling processes remains unclear. It has been reported that biochar-treated soil emissions are strongly dependent on biochar feedstock, pyrolysis method, soil type, soil water content and agricultural system^17–21^. Some research has revealed that reduced N_2_O emissions were accompanied by increased activity of *nosZ* gene bearing denitrifier microbial community in biochar amended soils^22–25^. More recent work described a shift of community composition of *nosZII* gene bearing bacteria under field conditions after biochar amendment, altering the relative abundance of specialized N_2_O reducers^26^.

Little research has been carried out to assess the effect on N_2_O emission of the simultaneous application of both NIs and biochar to N fertilized soils. Recent study of Keiblinger *et al*.^27^ reported the sorption of DMPP to soil-biochar mixtures at neutral pH, although these authors did not test the efficiency of DMPP in mitigating N_2_O emissions in biochar amended soils. Provided that both types of compounds have been proven to reduce N_2_O emissions, and the capacity of biochar to adsorb the DMPP molecules, the study´s objective was to reveal the influence of the nitrification inhibitor DMPP on N_2_O emissions in soils amended with biochar, as well as their combined effect on the nitrifying and denitrifying microbial populations responsible for N_2_O emissions under two contrasting soil water content conditions (40% and 80% WFPS).

## Materials and Methods

### Soil and biochar characterization

A silt loam soil was collected from the plough horizon (0 to 15 cm) of a typical grassland in the Basque Country of Northern Spain (43°17′23.2″N 2°52′20.2″W). The soil had a pH (1:2.5 H_2_O) of 6.0, a total C and total N content of 3.42 and 0.43%, respectively, and contained 2.2% coarse sand, 31.2% fine sand, 51.6% silt and 15.0% clay. Roots and stones were removed and the soil was sieved at 10 mm. The soil was then air-dried, homogenized and kept at 4 °C until used. Biochar was produced from Loblolly pine (*Pinus taeda*) chips pyrolyzed at 500 °C. The biochar was analyzed for its total C, N, H, S and O contents by ultimate analysis of coal following ASTM 3176-15 (Hazen Research Inc, Golden, CO USA; American Standard of Testing Material)^28^. The pH was measured in deionized water at a 1:2.5 biochar-water ratio. Moisture, ash and volatile matter contents were analyzed according to the standard test method for chemical analysis of wood charcoal (American Standard of Testing Material)^29^. Biochar particle size was determined through dry sieving^30^. Biochar physicochemical properties and elemental composition are summarized in Table 1.

**Table 1:** Physicochemical properties of the loblolly pine biochar.

*Loblolly pine* Biochar Characteristics	
Temp °C	500
pH	7.6
Moisture (%)	3.31
Ash (%)	3.74
Volatile (%)	24.00
Fixed C (%)	72.25
Sulfur (%)	0.002
Carbon (%)	80.03
Hydrogen (%)	3.17
Nitrogen (%)	0.56
Oxygen (%)	12.49
Particle summary (%):	
>2 mm	12.00
0.3–2 mm	77.63
0.075–0.3 mm	9.15
<0.075 mm	1.33

### Experimental incubation set up

The experimental design of the soil microcosm consisted of an arrangement in which six treatments were established by combining biochar and fertilizer application. The incubation study was performed simultaneously under two different moisture states and utilized 500 mL glass jars with 100 g of dry soil per jar. Main factor was biochar addition, with two levels: control without biochar and soil with biochar added at 2% (equivalent to 40 t ha^−1^). The control and biochar amended soils were subdivided into three groups according to the fertilizer treatment. The fertilizer groups were: unfertilized treatment, ammonium sulfate (AS 21%) and AS with DMPP, available in the market as ENTEC® 21 (EuroChem Agro Iberia S.L.). Ammonium sulfate was applied at a rate of 154 mg N kg^−1^ dry soil (equivalent to 180 kg N ha^−1^) to both AS treatments. In order to achieve homogenous fertilizer distribution in soil, AS was dissolved in deionized water and a 5 mL volume was applied with a pipette to the corresponding soils. For unfertilized treatments, 5 mL of deionized water were added. The resulting six treatments were assayed under two contrasting different moisture conditions expressed as soil water filled pore space (40% and 80% WFPS). Water was added to each jar to reach the moisture defined for each soil water content by the equation detailed in Aulakh *et al*.^31^. One set of jars (total n = 48) was used for repetitive N_2_O measurements over the course of the experiment (163 days), with four replicates per treatment. A second set of jars (total n = 144) was used for destructive sampling where four replicates per treatment and time point (days 11, 31 and 163) were sampled for NH_4_, NO_3_ and pH determinations, and three replicates out of these four were also sampled for soil nitrifying and denitrifying bacterial population determinations. To reactivate total soil microorganisms^32^, soil was rehydrated with deionized water up to 10% below the final WFPS and 0.5 g of glucose and 8.5 mg N per kg of dry soil (equivalent to 10 kg N ha^−1^) as ammonium sulfate nitrate (ASN) were added to each jar 15 days before treatments application. Jars were covered with parafilm with small perforations in order to avoid major water losses while maintaining aeration, and were incubated at 20 °C for 163 days. Once per week, jars were weighted and deionized water was added as necessary to adjust the WFPS.

### N_2_O emissions

Soil N_2_O emissions were measured in the four replicates per treatment two or three times per week during the first month after treatments application. Afterwards, measurements were continued at a frequency of once per week until the end of the experiment. For each measurement, the jars were hermetically sealed with a lid supporting a butyl rubber septum and 20 mL of the inner headspace air were collected using a gas-tight syringe at 0, 45 and 90 min after sealing the jars. Gas samples were stored at overpressure in pre-evacuated 12 mL glass vials and analyzed using a gas chromatograph (GC, Model 7890A, Agilent Tech., USA), equipped with an electron capture detector (ECD) to quantify N_2_O. A capillary column (IA KRCIAES 6017: 240 °C, 30 m × 320 μm) was used and the samples were injected by means of a headspace autosampler (Teledyne Tekmar HT3) connected to the GC. Standards of N_2_O were analyzed at the same time as the samples. Fluxes were calculated on a daily basis from the linear increase in concentration in the jars headspace over the 90 minute incubation time^33^. Cumulative N_2_O emissions were estimated by multiplying the average of two consecutive measurements by the time period between those measurements and adding that amount to the previous cumulative value.

### Soil ammonium, nitrate and pH analyses

Evolution of soil ammonium, nitrate and pH were determined in the four replicates per treatment at each time point. In order to determine soil NH_4_^+^ and NO_3_^−^, 100 g of dry soil were mixed with 1 M KCl (1:2, w:v) and shaken for 1 h at 165 rpm. The soil solution was filtered through Whatman no. 1 filter paper (GE Healthcare, Little Chalfont, Buckinghamshire, UK) and then through a Sep-Pak Classic C18 Cartridge (125 Å pore size; Waters, Milford, MA, USA) to eliminate organic carbon. Nitrate content was determined as described by Cawse^34^ and NH_4_^+^ content by the Berthelot method^35^. For soil pH determination, 10 g of dry soil were suspended in deionized water (1:2.5, w:v) and shaken for 1 h at 165 rpm. Soil suspensions were then settled for 30 min and pH was determined in the solution phase.

### DNA isolation and quantification of nitrifying and denitrifying gene abundance

Quantitative polymerase chain reaction (qPCR) was used to quantify the abundance of microbial nitrogen-cycling functional marker genes. Soil DNA was isolated from three of the four same replicates used for NH_4_ and NO_3_ determination. Soil samples were mechanically homogenized for 3 min in a ball-mill grinder (Mixer Mill MM 400, Retsch, Haan, Germany) and then stored at −80 °C until analysis. Total DNA was extracted from 0.25 g of soil using the PowerSoil DNA Isolation Kit (MO BIO Laboratories, Carlsbad, CA, USA) with the following modifications: cell lysis was carried out in a homogenizer Precellys24 (Bertin, Montigny-le-Bretonneux, France), cooling incubations and final elution incubation was performed as described by Harter *et al*.^24^. Soil DNA concentration and quality were determined spectrophotometrically (NanoDrop 1000, Thermo Scientific, Waltham, MA, USA).

Quantification of bacteria and archaea abundances (16S rRNA) and functional marker genes involved in nitrification (*amoA*) and denitrification (*narG*, *nirK*, *nirS*, *nosZI* and *nosZII*) were amplified by qPCR using SYBR® Premix Ex Taq™ II (Takara-Bio Inc.) and gene-specific primers (Supplemental Table 1). Each sample was quantified in triplicate using the StepOnePlus™ Real-Time PCR System and data analysis was performed by StepOnePlus™ Software v2.3 (Thermo Scientific). Standard curves were prepared from serial dilutions of 10^7^ to 10^2^ gene copies μl^−1^ linearised p-GEMT plasmids with insertions of target gene fragments (Promega Corporation, Madison, WI, USA), following the equations detailed in Torralbo *et al*.^13^. The copy number of target genes per gram of dry soil was calculated according to a modified equation detailed in Behrens *et al*.^36^: [(number of target gene copies per reaction X volume of DNA extracted)/(volume of DNA used per reaction X gram of dry soil extracted)]/DNA concentration.

### Statistics

All results are reported as mean ± standard deviation. Data sets were analyzed for normality of residuals and homogeneity of variances using Kolmogorov-Smirnoff. The data that did not pass normality test were transformed. Significant differences between treatments were analyzed using multi-factorial repeated measures ANOVA followed by Fisher’s LSD test. The effect size of each factor and their interactions was determined with the use of partial eta-squared, which describes a proportion of variability in a sample associated with an independent variable: ƞ^2^_p_ = SS_effect_/(SS_effect_ + SS_error_), where SS_effect_ is the sum of squares for the effect of interest and SS_error_ is the error term associated with this effect effect^37^. Relationships between different variables were tested by Spearman’s correlation. Additional details and significance levels are described in the figures captions. All analyses were performed at a significance level p < 0.05 by the Statistica package (Dell Inc, Round Rock, Texas, USA).

## Results

### N_2_O emissions

**Figure 1:**
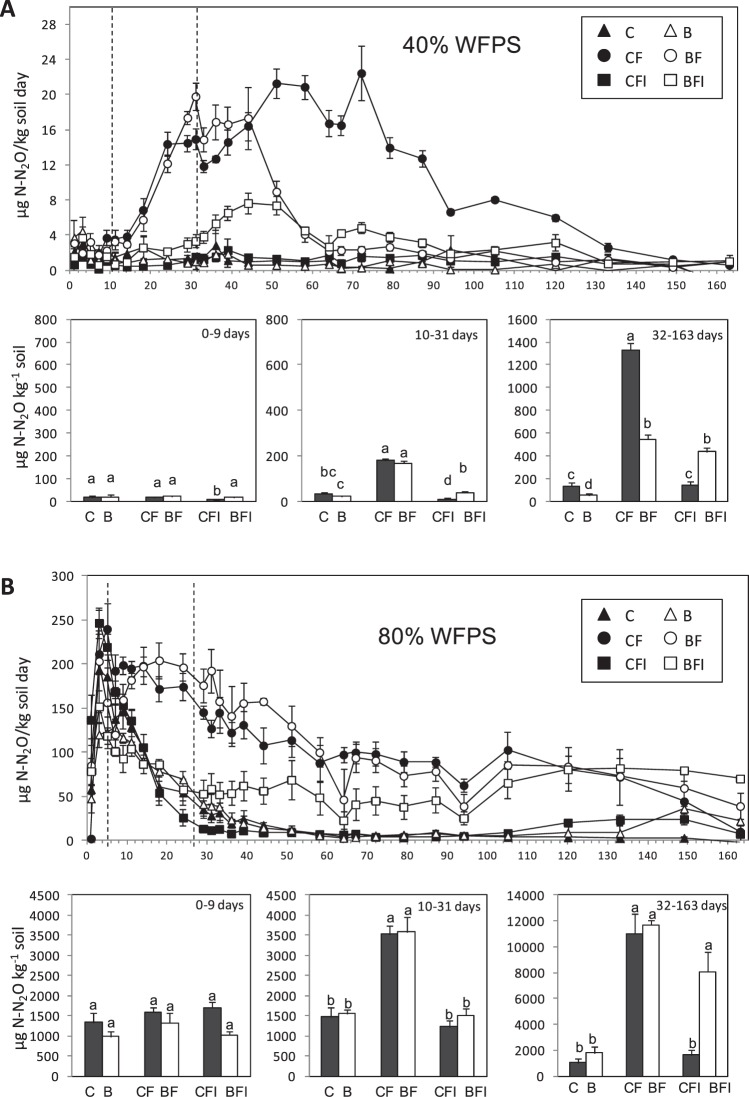
N_2_O emission rates at 40% (**A**) and 80% (**B**) of WFPS and N_2_O cumulative emissions for the three different periods at each soil water content in the control (black bars) and 2% (w/w) biochar-containing soil (white bars). Symbols: triangle unfertilized, circle fertilized with AS and square fertilized with AS + DMPP. Black symbols are control soils and white symbols are biochar amended soils. Treatments sharing the same letter within each period do not differ significantly at p ≤ 0.05. Dotted lines show separation between periods.

As shown in Table 2, the three factors assayed (WFPS, biochar addition and fertilization treatment) significantly affected N_2_O emissions, and interactions between these factors were also observed. Partial ƞ^2^ values indicate that most of the effect on N_2_O emissions was due to WFPS, followed by fertilizer treatment and into a lesser extent by to biochar addition. The cumulative N_2_O emission rates for each treatment across the entire experimental period are shown in Fig. 1. Over the 163 day experiment, three different periods were distinguished on the basis of the observed DMPP inhibitory capacity on N_2_O emissions. First, a *lag* phase from 0 to 9 days; a second phase from 10 to 31 days where DMPP reduced N_2_O emissions down to unfertilized levels; and a third phase from 32 to 163 days where the efficiency of DMPP reducing N_2_O emissions in biochar amended soils (BFI) decreased compared to the non-biochar amended control soils (CFI).

**Table 2:** Significance and size effect of each factor (WFPS, biochar and fertilizer) and their interactions on the different variables measured.

	N_2_O		pH		NO_3_^−^		NH_4_^+^		Bacteria	
	Sig.	partial ƞ^2^	Sig.	partial ƞ^2^	Sig.	partial ƞ^2^	Sig.	partial ƞ^2^	Sig.	partial ƞ^2^
WFPS (%)	***	0.993	*	0.253	***	0.673	***	0.598	*	0.354
biochar (%)	*	0.219	*	0.266	*	0.269	***	0.966	n.s.	0.218
fertilization	***	0.821	n.s.	0.060	***	0.757	***	0.747	n.s.	0.240
WFPS (%)*biochar (%)	**	0.329	*	0.322	**	0.352	***	0.538	n.s.	0.087
WFPS (%)*fertilization	***	0.355	**	0.246	**	0.275	***	0.559	n.s.	0.216
biochar (%)*fertilization	***	0.441	***	0.329	**	0.251	***	0.348	n.s.	0.135
WFPS (%)*biochar (%)*fertilization	**	02.47	***	0.386	n.s.	0.100	***	0.508	n.s.	0.106
	**Archaea**		**AOB**		**AOA**		***nosZI***		***nosZII***	
WFPS (%)	***	0.888	***	0.689	***	0.952	***	0.689	***	0.874
biochar (%)	n.s.	0.037	***	0.575	**	0.466	n.s.	0.161	**	0.459
fertilization	***	0.694	***	0.680	**	0.366	***	0.499	**	0.331
WFPS (%)*biochar (%)	n.s.	0.171	n.s.	0.171	n.s.	0.089	n.s.	0.268	**	0.420
WFPS (%)*fertilization	***	0.625	**	0.379	***	0.547	***	0.444	**	0.357
biochar (%)*fertilization	***	0.436	***	0.492	**	0.382	n.s.	0.207	n.s.	0.203
WFPS (%)*biochar (%)*fertilization	n.s.	0.217	**	0.327	*	0.254	n.s.	0.123	n.s.	0.045
	***nirS***		***nirK***		***narG***		**AOA**/**AOB**		**(*nosZI ***+*** nosZII*)/(nirS + nirK)**	
WFPS (%)	***	0.912	n.s.	0.101	**	0.403	***	0.928	**	0.504
biochar (%)	n.s.	0.294	*	0.392	n.s.	0.176	***	0.670	*	0.386
fertilization	***	0.661	*	0.240	***	0.497	***	0.787	***	0.476
WFPS (%)*biochar (%)	n.s.	0.148	n.s.	0.045	n.s.	0.102	n.s.	0.162	n.s.	0.139
WFPS (%)*fertilization	***	0.671	***	0.388	***	0.517	***	0.627	**	0.399
biochar (%)*fertilization	**	0.375	n.s.	0.179	n.s.	0.214	***	0^,.^509	*	0.266
WFPS (%)*biochar (%)*fertilization	n.s.	0.146	n.s.	0.158	*	0.268	*	0.287	n.s.	0.113

*p < 0.05, **p < 0.01, ***p < 0.001. Partial η^2^ describes proportion of the total variability attributed to a factor (Levine & Hullett 2002).

When cumulative emissions of each period were analyzed, the *lag* phase showed no effect of fertilizer, DMPP or biochar on N_2_O emissions regardless of soil water content. In the second period, the AS application (CF) significantly increased N_2_O emissions at both soil water contents, as compared to the unfertilized soil (C), while DMPP(CFI) reduced N_2_O emissions comparable to unfertilized soils at both 40% and 80% WFPS conditions. This reduction was of about 85% at low soil water content and of 62% at high soil water content with respect to the fertilized treatments. No clear effect was observed in relation to biochar application during the second phase. In the third period, reductions of 89% and 85% on N_2_O emissions were observed at low and high WFPS when DMPP was applied as compared to the fertilized treatments, while no reduction was observed when DMPP was added to biochar amended soils (BFI) at 40% of WFPS and a 31% reduction of N_2_O emissions was observed at 80% of WFPS as compared to fertilized treatments (BF). In fact, the efficiency of DMPP mitigating N_2_O emissions decreased in the biochar amended soil from approximately day 31 onwards at the 40 and 80% WFPS. Regarding the effect of biochar alone on N_2_O emissions during this last period, a significant (p < 0.05) reduction of 60% was observed in the fertilized biochar amended soil (BF) with respect to the fertilized control soil (CF) at 40% WFPS from day 45 onwards, while no effect was observed at 80% of WFPS.

### Soil ammonium, nitrate, and pH evolution

**Figure 2:**
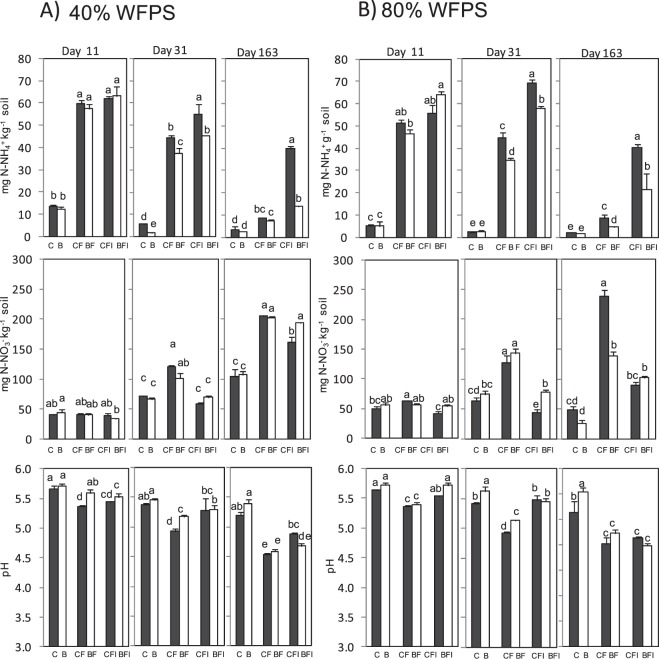
Soil ammonium and nitrate contents (top and middle) and soil pH values (bottom) at the end of the three periods along the incubation experiment in the control (black bars) and 2% (w/w) biochar-containing soil (white bars) at 40% (**A**) and 80% (**B**) of WFPS. Treatments sharing the same letter within each day do not differ significantly at p ≤ 0.05. Fisher’s LSD post-hoc test was performed for each variable, except for nitrate on day 163 when, due to low statistical power, pairwise comparisons after Kruskal-Wallis were employed.

Soil ammonium and nitrate contents were analyzed at the end of each of the three periods, on days 11, 31 and 163 (Fig. 2). Both parameters were affected by WFPS, fertilization and biochar amendment, while the effect size of each factor was different. The effect of WFPS and fertilization on soil nitrate and ammonium contents was similar in size (Table 2). On the other hand, the proportion of variance explained by biochar amendment was greater for soil ammonium content than for nitrate. Soil pH remained unaffected by fertilization, and the effect size of WFPS and biochar addition was low. The AS application increased soil ammonium content with respect to non-fertilized soils, as expected (Fig. 2). At the end of the *lag phase*, on day 11, little changes were observed in nitrate contents. A trend to diminish soil pH was observed when fertilizer was applied, more significantly under high soil water content. The addition of DMPP ameliorated this acidification effect at 80% WFPS, maintaining unfertilized soil pH values in the BFI.

By the end of the second phase, on day 31, a significant decrease in ammonium concentration was observed at both soil water contents in biochar amended soils for all fertilized treatments. A net increase of the nitrate content was observed at both WFPSs in fertilized soils CF and BF, possibly due to the nitrification of ammonium to nitrate. Treatments with DMPP (CFI and BFI), where ammonium oxidation was avoided, showed higher ammonium contents and lower nitrate contents than the fertilized ones without inhibitor (CF and BF). At 80% WFPS there were no differences in soil nitrate content between the control soil and the biochar amended soil when fertilizer was applied. However, at 40% WFPS the biochar amended soil showed a slightly lower soil nitrate content. Indeed, the reduction observed in soil nitrate content when DMPP was applied at 80% WFPS was ameliorated when soils were biochar amended, since they showed higher nitrate contents than the control soils with DMPP. Soil pH values decreased half a unit (from 5.5 to 5) in unfertilized soils to soils receiving AS (Fig. 2). In the case of biochar amended soils, this acidification occurred to a lesser extent. The application of AS combined with DMPP avoided soil acidification, maintaining the same soil pH values of the unfertilized soils. However, in biochar amended soils, the DMPP effect on ameliorating acidification was less pronounced than without biochar, not maintaining the same values of the unfertilized soils.

By the end of the incubation period on day 163, the soil ammonium concentration of the fertilized treatments (CF and BF) had decreased to almost that of the unfertilized soils at both soil water contents. However, due to avoided nitrification, the soil ammonium content remained much higher when DMPP was applied to fertilized control soils (CFI). On the contrary, in biochar amended soil the ammonium content was decreased. A clear net increase of nitrate was observed in the fertilized treatments CF and BF, possibly due to nitrification. However at 80% WFPS the nitrate content was lower in BF. When DMPP was applied, at 40% WFPS BFI reached the same nitrate level as BF, while CFI remained lower than CF. At high soil water content both soils amended with DMPP (CFI and BFI) maintained lower nitrate levels than the fertilized ones without inhibitor. Soil pH values remained stable in unfertilized soils and decreased in soils receiving AS, at both soil water contents and in both control and biochar amended soils (Fig. 2). At the end of the experiment, the effect of DMPP addition was dependent on soil water content, since at 40% WFPS an amelioration of soil acidification was observed due to DMPP application while at 80% WFPS soil pH values remained as low as in the fertilized soils, both in control and biochar amended soils.

### Abundance of 16S rRNA and N-cycle functional marker genes

**Figure 3:**
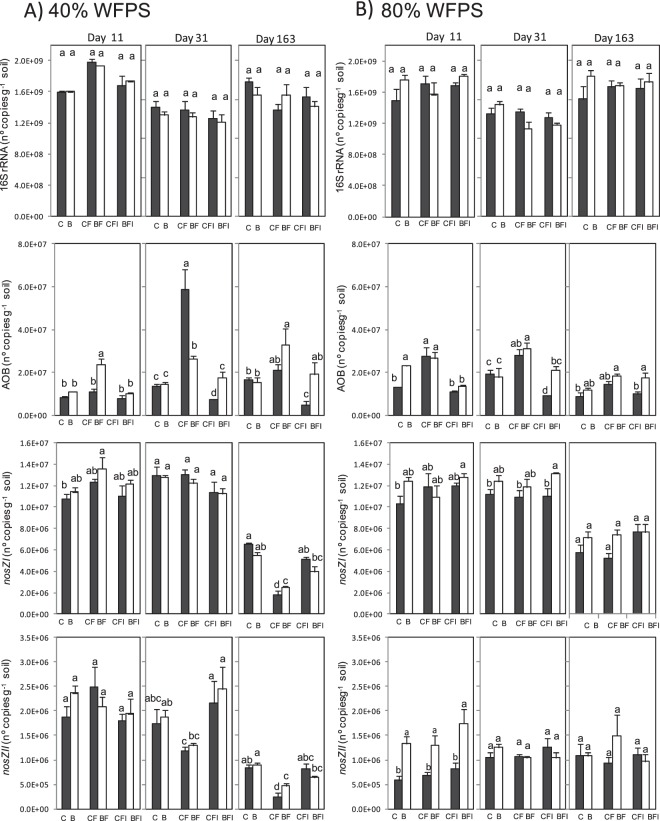
Gene copy numbers per gram dry soil over time for various key genes of microbial nitrogen transformation processes in the control (black bars) and 2% (w/w) biochar-containing soil (white bars) at 40% (**A**) and 80% (**B**) of WFPS. From top to bottom panel summarizes the gene copy numbers for 16S rRNA, *amoA*, *nosZI* and *nosZII*. Treatments sharing the same letter within each day do not differ significantly at p ≤ 0.05.

Total bacterial abundance, measured as 16S rRNA gene abundance, remained stable independently of the experimental factor assayed (Table 2) with only slight changes with WFPS and over incubation time, fluctuating between 1.1 × 10^9^ and 1.9 × 10^9^ gene copies per g dry soil (Fig. 3). Contrary, changes in the ammonia oxidizing bacteria (AOB) abundance, measured as *amoA* gene copy numbers, were registered concomitantly to the dynamics observed in N_2_O emissions and soil inorganic N content. Therefore, WFPS, biochar and fertilization treatments significantly affected the AOB population, with all three factors showing similar effect sizes (Table 2). During the first eleven days at 40% WFPS there were no or little changes in *amoA* gene copy number, except being two times greater in BF with respect to the other treatments (Fig. 3). At high soil water content, unfertilized biochar amended soil (B) and both fertilized soils CF and BF showed higher amounts of *amoA* gene compared to other treatments. At the end of the second phase, a significant increase in the AOB abundance was observed at 40% WFPS when AS was added, while AOB was below the unfertilized treatment when DMPP was applied, leading to an 86% lower value in CFI with respect to CF (Fig. 3). Similar trends were observed at 80% WFPS, with AOB increasing with AS application while being maintained or even decreased to below the unfertilized base levels when DMPP was applied. Biochar applied with AS reduced AOB by 55% as compared to AS alone on day 31 (BF vs CF) at 40% WFPS, according to the mitigation of 60% observed in N_2_O emissions in the following third period of incubation (Fig. 1). At 80% WFPS biochar amendment did not change the AOB population in BF with respect to CF. When DMPP was applied in biochar amended soils (BFI) no reduction in AOB was observed at 40% of WFPS and a reduction of only 32% was observed at 80% WFPS as compared to AS biochar amended soils (BF). As a result, the inhibitory effect provoked by DMPP alone on the AOB population was buffered when biochar was present, leading up to a population of AOB which was double in BFI with respect to CFI. By the end of the experiment on day 163, when DMPP was applied to fertilized soils (CFI), a 78% lower AOB population was still maintained at 40% WFPS as compared to fertilized soil (CF), while at 80% WFPS no significant reduction was observed. The reduction of AOB was only of 42% when DMPP was applied in biochar amended soil (BFI) at 40% WFPS, and no reduction was observed at 80% WFPS.

The abundance of nitrous oxide-reducing bacteria was measured as *nosZI* (also called typical *nosZ*) *and nosZII* (atypical *nosZ*) genes copy numbers, being both mostly affected by WFPS (Table 2). The *nosZI* gene abundance did not show any significant effect of treatments at the end of the first phase of the incubation period (Fig. 3) and only a slight trend to increase in biochar amended soils at 80% WFPS at the end of the second phase. At the end of the incubation period on day 163, *nosZI* gene copy numbers were around three and two times lower at 40% and 80% WFPS respectively in relation to day 31. A significant decrease of *nosZI*-denitrifiers was registered in fertilized treatments at 40% WFPS with respect to the unfertilized soil, a decrease that was ameliorated when DMPP was applied in both control and biochar amended soils. At 80% WFPS, although being no significant, a trend to increase *nosZI* values was observed in biochar amended soils except for soil receiving also DMPP.

*NosZII*-denitrifiers were less abundant than *nosZI*-denitrifiers. At the end of the *lag* phase no differences between treatments were observed at 40% WFPS, but higher amounts of *nosZII* gene were registered in all biochar amended soils at 80% of WFPS compared to all treatments not receiving biochar. During the rest of the experiment, a significant decrease of *nosZII* gene abundance was registered with time as well as with the application of fertilizer at 40% WFPS, while the decrease provoked by fertilizer was avoided by the addition of DMPP in control and to a lesser extent in biochar amended soils. This effect was not observed at high soil water content.

**Figure 4:**
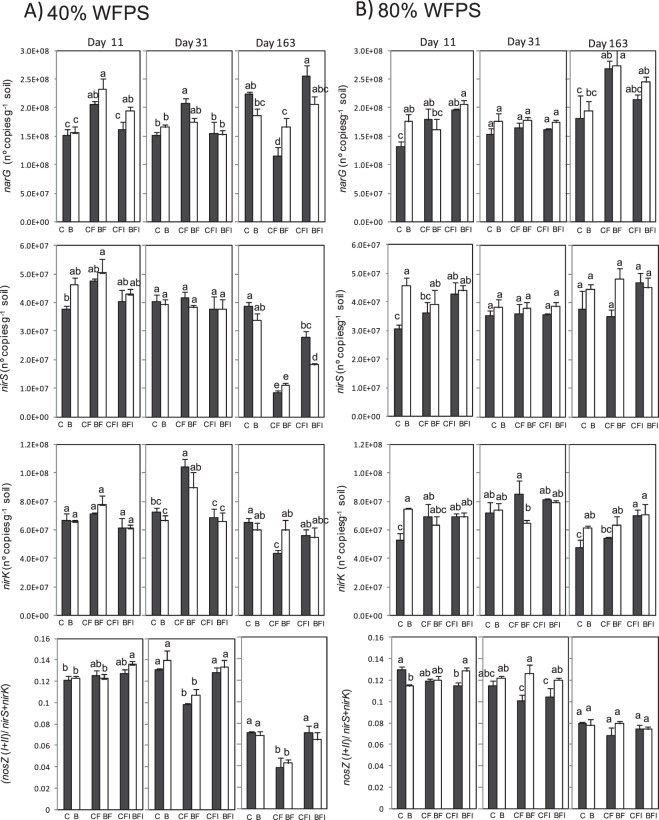
Gene copy numbers per gram dry soil over time for various key genes of microbial nitrogen transformation processes in the control (black bars) and 2% (w/w) biochar-containing soil (white bars) at 40% (**A**) and 80% (**B**) of WFPS. From top to bottom panel summarizes the gene copy numbers for *narG*, *nirS*, and *nirK*, and the ratio of *nosZ* genes over the sum of *nirS* and *nirK* genes copy numbers (*nosZI* + *nosZII*/*nirK* + *nirS*). Treatments sharing the same letter within each day do not differ significantly at p ≤ 0.05.

The remainder of genes involved in denitrification showed a slight or no response to biochar addition, and a varied response to WFPS and fertilization (Table 2). By the end of the *lag* phase it was remarkable that at 80% of WFPS all denitrifying genes (*narG*, *nirS* and *nirK*) showed higher copy numbers in biochar amended soil when no fertilizer was added (Fig. 4). At the end of the experiment on day 163, the decrease observed in *nirS* gene abundance at 40% WFPS in fertilized soils as compared to unfertilized soils, was partly avoided when DMPP was added. Again, this amelioration effect was lower in the biochar amended soil. A similar trend, although to a lesser extent, was observed for *narG* gene abundance. The ratio of the sum of *nosZI* and *nosZII* gene copies over the sum of *nirK* and *nirS* gene copies (*nosZI* + *nosZII*/*nirS* + *nirK*) illustrates proportions between nitrous oxide-reducing bacteria and nitrite-reducing bacteria, suggesting a shift in the N_2_ versus N_2_O production ratio in the denitrification process. This ratio values were always below 0.14, which means that nitrite-reducing bacteria were more abundant whatever the treatment. However DMPP (CFI and BFI) avoided the decrease in this ratio provoked by AS fertilization (CF and BF) on days 31 and 163 at 40% WFPS (Fig. 4).

**Figure 5:**
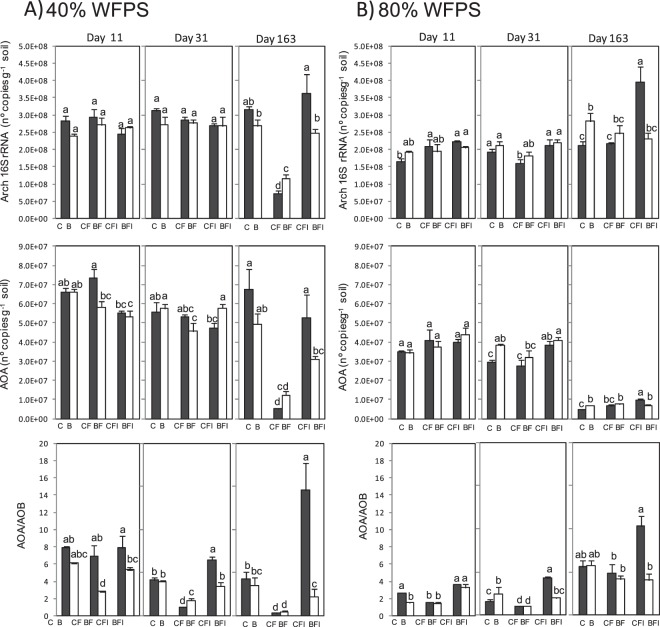
Gene copy numbers per gram dry soil over time for archaeal genes in the control (black bars) and 2% (w/w) biochar-containing soil (white bars) at 40% (**A**) and 80% (**B**) of WFPS. From top to bottom panel summarizes the gene copy numbers for archaeal 16S rRNA, archaeal *amoA* (AOA) and the ratio of AOA over AOB (AOA/AOB ratio). Treatments sharing the same letter within each day do not differ significantly at p ≤ 0.05.

Total soil archaeal abundance, measured as archaeal 16S rRNA gene abundance, fluctuated around 2.5 × 10^8^ gene copies per g dry soil during the incubation period, a six-fold lower amount than bacterial abundance (Fig. 5). In general, the archaeal population did not change with biochar amendment (Table 2), with WFPS being the factor with the highest effect size. Fertilization treatment influenced the archaeal population, markedly on day 163 at 40% of WFPS, when AS application led to a significant decrease compared to other treatments (Fig. 5). These changes were reflected in AOA gene copy number, which followed the same pattern. The effect of DMPP decreasing AOB on the one hand, and ameliorating the decrease in AOA on the other (CFI), led to a final rise of the ratio AOA/AOB at the end of the incubation period at both low and high soil water contents as compared to fertilized treatment (CF) (Fig. 5). However, the application of DMPP in biochar amended soil (BFI) did not increase the AOA/AOB ratio. This was due to a buffered effect of DMPP in biochar amended soils, preventing both the AOB decrease (BFI vs BF, Fig. 3) and avoiding AOA recuperation up to unfertilized levels (Fig. 5).

## Discussion

### DMPP efficiency reducing N_2_O emissions

The nitrification inhibitor DMPP has been shown to reduce N_2_O emissions under different climatic and soil conditions^10,38–40^, with in-field N_2_O emission reduction efficiencies ranging from 0% to 60%^8,9,41–44^. In our experiment, the efficiency of DMPP was higher than 85% at both soil water contents, and DMPP managed to reduce N_2_O cumulative emissions down to the unfertilized soil levels (Fig. 1), concomitantly with a decrease in AOB populations, higher NH_4_^+^ and lower NO_3_^−^ soil contents (Figs 2 and 3); this effect was observed at both soil water contents. It is accepted that N_2_O emissions are closely related to soil water content^45^ and that 60% WFPS appears to be the threshold between water-limited and aeration limited microbial processes in a wide range of soils. In our work, the high value of partial eta-squared for WFPS (0.993, Table 2) evidences the strong impact of soil water content on N_2_O emissions. In fact, N_2_O emission rates were ten times greater at 80% than at 40% WFPS. Under relatively high soil water content conditions, denitrification is expected to be the dominating process responsible for N_2_O production. Nevertheless, our data demonstrate that DMPP effectively reduced N_2_O losses under high soil water content, suggesting that nitrification was also taking place. According to Hoffmann *et al*.^46^, the intensity of nitrification is influenced by soil structure, which regulates aeration. Using a previously broken and sieved soil should have enhanced O_2_ availability at 80% WFPS, allowing both nitrification and denitrification to occur simultaneously. The simultaneous occurrence of both processes has been described previously^47,48^ and can be explained due to aerobic and anaerobic microsites existing within the same soil aggregates. In this sense, several authors^13,32,49^ have reported that nitrification can occur at WFPS values around 85% in different soil types. Moreover, an induction of AOB was observed at 80% WFPS when AS was applied, although two times lower than at 40% WFPS (Fig. 3). Contrarily to other studies where the AOB population was unaffected at high soil water content^11^, DMPP showed a high efficiency mitigating N_2_O emissions at both soil water contents via a direct effect on nitrification, since AOB abundance was significantly reduced at both conditions.

The only change observed in nitrifying archaea with AS application was via a decrease of total soil archaeal population (Fig. 5), since DMPP usually does not affect *amoA* of ammonia oxidizing archaea^13,40,50,51^. It is well known that nitrogen fertilization usually has a negative impact on soil archaeal populations. In fact, the activity and growth of AOB are favored in nutrient rich, high N soil, whereas AOA may rather proliferate under nutrient-poor, low N conditions^52,53^. By the end of the experiment, the nitrifying bacteria growth inhibition exerted by DMPP caused a lower competition between nitrifying bacterial and archaeal populations, leading thus to an increase in the AOA/AOB ratio (Fig. 5).

**Figure 6:**
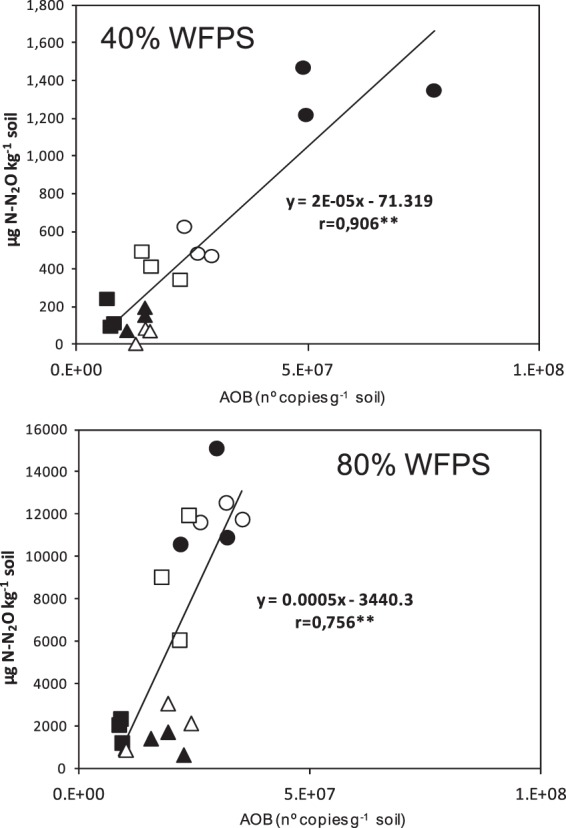
Simple regression analysis between cumulative N_2_O emissions during the third phase and ammonia oxidizing bacterial populations determined in the different treatments on day 31 at 40% (top) and 80% (bottom) WFPS (n = 18). Symbols: triangle unfertilized, circle fertilized with AS and square fertilized with AS + DMPP. Black symbols are control soils and white symbols are biochar amended soils. **Significant at p < 0.01.

Like many other grassland soils^53–55^ AOA numerically dominated over AOB in the silt loam soil used in this study. However, previous works revealed that the numerical advantage at genomic level does not necessarily equal the dominance at functional level^56^ a reason why changes in AOB populations could explain the mitigation effect exerted by DMPP on N_2_O emissions. In fact, a high and positive correlation between the AOB abundance at the end of the second phase, on day 31, and the cumulative N_2_O emission of the following third phase was found at both soil water contents (r = 0.906 and r = 0.756 at 40% and 80% WFP respectively) (Fig. 6). This indicated that the AOB population present in the soil at the end of the second phase, at day 31, seemed to be functional and directly or indirectly responsible for the N_2_O emissions of the following last phase of the experiment.

**Figure 7:**
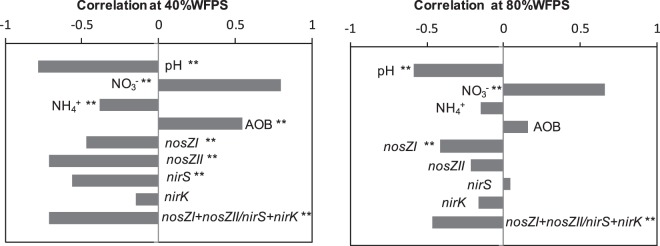
Spearman correlation coefficients between cumulative N_2_O emissions of each three phases and their corresponding physicochemical/microbial factors at 40% (left) and 80% (right) WFPS (n = 54). *Significant at p < 0.05, **significant at p < 0.01.

Several studies have described that recommended field application rates of nitrification inhibitors, such as DMPP, do not affect non-target soil microbial metabolism^9,57–59^. This was also demonstrated in the present study by means of the unchanged abundance of the 16S rRNA gene (Fig. 3). However, as suggested by previous studies^11,13^, dimethylpyrazole-based inhibitors can affect not only the nitrification process but also the denitrification process. Torralbo *et al*.^13^ described an increase in the abundance of both typical and atypical *nosZ* genes in the presence of DMPP, yet this effect was observed only at high soil water content. In our case, changes regarding denitrifying bacteria were observed at both low and high soil water contents, although greater at 40% WFPS (Fig. 3). Related to this, changes in soil pH can influence soil denitrifying microorganisms. Low soil pH interferes with the assembly of the enzyme nitrous oxide reductase, and posttranscriptional negative effect of low pH on the expression of this enzyme in soil bacterial communities have been demonstrated^60^. In our experiment, the addition of DMPP to soils significantly ameliorated the acidification produced due to AS application (Fig. 2), and denitrifying soil populations responded to this soil pH recovery, as demonstrated by the highly significant correlation between soil pH and the (*nosZI* + *nosZII*/*nirS* + *nirK*) ratio (r = 0.598, p < 0.01). The decrease observed in both *nosZ* genes bearing denitrifying bacteria at low soil water content when AS was applied was ameliorated with the application of DMPP. This implies that, although nitrification was supposed to be a prevailing process at 40% WFPS, denitrification was also occurring, probably due to the coexistence of aerobic and anaerobic microsites in the soil as previously discussed. So, DMPP could affect this denitrification process at 40% WFPS inducing changes in *narG*, *nirS* and *nosZ* gene copy numbers (Figs 3 and 4) leading to a net increase in the (*nosZI* + *nosZII*/*nirS* + *nirK*) ratio, which implies the potential for a specific induction of N_2_O reduction to N_2_ in DMPP treated soils. In this sense, identifying and developing agricultural practices, such as the use of NIs, that promote complete denitrification through stimulation of exclusive N_2_O reducers might be one way to reduce N_2_O emissions and a step towards climate smart agriculture^26^. In fact, negative correlations were found between N_2_O emissions and the (*nosZI* + *nosZII*/*nirS* + *nirK*) ratio at both soil water content conditions (r = −0.713 and r = −0.461 at 40% and 80% WFPS, respectively) (Fig. 7). According to Jones *et al*.^61^ and Orellana *et al*.^62^, about half of the atypical nosZ gene containing microorganisms do not carry the functional genes encoding nitrate, nitrite and nitric oxide reductases and are thus only capable of reducing N_2_O to N_2_, behaving as sinks of N_2_O produced by other microorganisms. In our soil, typical *nosZ*-bearing bacteria were more abundant than atypical *nosZ*-bearing ones. Nevertheless, at 40% WFPS both increased in response to DMPP, contributing to the mitigation of N_2_O emission. At 80% WFPS, DMPP application also induced an increase in *nosZI* gene copy number, while no other changes were registered in the rest of the key genes of denitrification. This is in agreement with Barrena *et al*.^11^ and Torralbo *et al*.^13^, whose works described an induction of *nosZ*-denitrifiers, although these authors observed induction only at high soil water content. Soil nitrate content was also highly correlated with the (*nosZI* + *nosZII*/*nirS* + *nirK*) ratio at both WFPS conditions (r = −0.887 and r = −0.411; p < 0.001 at 40% and 80% WFPS, respectively). This is fully in agreement with the well-known fact that higher nitrate concentrations usually result in higher N_2_O:N_2_ ratios in the denitrification process because of an incomplete denitrification due to suppression of nitrous oxide reductase activity^63^. Provided that NIs prevent the conversion of ammonium to nitrate, the lower nitrate content induced after NIs application can consequently can, besides reducing N_2_O emissions from nitrification, also reduce N_2_O emissions from denitrification.

### Biochar efficiency in reducing N_2_O emissions

The mechanisms responsible for the mitigation of soil N_2_O emissions induced by biochar amendment remain elusive^63^. These mechanisms will most likely be a function of both biochar and soil properties and their specific interactions. The biochar used in this study had a C:N ratio of 129, largely surpassing the value of 30 described by Cayuela *et al*.^14^ as the value from which biochar could potentially reduce N_2_O emissions. Although in our experiment the effect of biochar amendment resulted was less significant and smaller than those WFPS or fertilization, the significant interaction between factors (Table 2) indicated that the mitigation in N_2_O emissions observed at 40% WFPS after biochar addition (Fig. 1) was significant. Biochar amendment to soils alter numerous geochemical parameters (e.g., nitrogen speciation, nutrient availability, pH and oxygen saturation) and these alterations indirectly affect the diversity, abundance and functioning of N_2_O-producing microbial communities in soils and concomitantly soil N_2_O emissions^3^. Changes in soil properties could influence N mineralization-immobilization, turnover and nitrification or denitrification processes. Increased soil pH following biochar application has been extensively investigated in agricultural soils and generally depends on the biochar pH, soil pH, ash content and soil buffering capacity^64–66^. The biochar used in this experiment was not highly alkaline, with a pH of 7.6 while the soil pH was 6.0. Given the biochar application rates used, it was not surprising that soil pH was not altered significantly. Thus, biochar’s effect on pH likely did not influence N_2_O production in this experiment, since the effect size of biochar amendment on soil pH was low (Table 2).

Biochar addition explained a great proportion of the variance of soil ammonium content (ƞ^2^_p_ = 0.966), significantly and consistently decreasing the extractable NH_4_^+^ content across fertilizer treatments and soil water contents (Fig. 2). Ammonium retention by biochar may be readily explained by electrostatic adsorption to negatively charged oxygen-containing surface functional groups^67,68^ or the negatively charged molecular orbitals of the aromatic carbon fractions. Singh *et al*.^69^ also described that the addition of biochar to soils increases sorption of inorganic nitrogen compounds such as NH_4_^+^ and NO_3_^−^ which decreases their availability for microbial N_2_O production. Additionally, NH_4_^+^ can react directly with CO_2_, further complicating our ability to discern the active process^70^. Although the exact mechanism for NH_4_^+^ retention has not been identified, it has been suggested that physical entrapment of NH_4_^+^ in biochar pore structures may be responsible^71^. The decrease we observed in NH_4_^+^ content consequently caused a decrease in nitrification and subsequently in NO_3_^−^ content, and could be the reason for the 60% decrease in N_2_O emissions observed at 40% WFPS in the biochar amended soil compared to the control (Fig. 1). Under high soil water content conditions this mitigating effect was not observed.

Biochar addition did not alter the soil total bacterial or archaeal abundances, but did exert an effect on the nitrifier community depending on the soil moisture. At 40% WFPS, a decrease of 53% in the AOB population occurred with respect to the control soil when AS was added, while at 80% WFPS biochar did not affect the AOB population (Fig. 3). The lower AOB abundance at low water content correlated with decreases in soil ammonium and nitrate contents (Fig. 2), as well as with the decrease in cumulative N_2_O emissions (Fig. 1). Thus, biochar may potentially reduce N_2_O emissions by affecting ammonia-oxidizing bacteria at relatively low soil moisture contents. Highly significant correlations were observed between N_2_O emission and AOB abundance (Fig. 6), and between N_2_O emissions and soil nitrate content (Fig. 7) under these conditions. Lower nutrient accessibility in the biochar pores than in natural soil pores reduces AOB production, and leads to less NO_3_^−^ produced by nitrification. On the other hand, at high soil water contents biochar pores may become clogged, thus limiting its adsorption capacity^72^. In this situation, both nitrifiers and denitrifiers have accessibility to nutrients and they could develop normally. This could explain why biochar alone did not mitigate N_2_O emissions at 80% of WFPS. Likewise, it has been demonstrated in both incubation^23–25^ and field experiments^26^ that reduced N_2_O emissions after biochar addition was accompanied by an increased abundance or activity of *nosZ*-bearing denitrifiers and a shift in denitrifier community composition. However, it has been suggested that denitrifying conditions are a prerequisite to this effect^26,73^. Harter *et al*.^25^ found that biochar addition to a fertilized, water saturated microcosm increased typical *nosZ* gene copy numbers whereas atypical *nosZ* gene copy numbers were not affected. In our case, under low soil water content conditions biochar did not exert any effect on denitrifier populations (Figs 3 and 4). Biochar led to an increase in *nosZI*-bearing denitrifiers in fertilized soils at high moisture, which is in agreement with previous studies^23–25,73,74^. Besides, we found an increase in *nosZII*-bearing denitrifiers in all biochar amended soils at the beginning of the experiment. However contrarily to previous findings, this increase in typical and atypical *nosZ* genes was not accompanied by a general decrease in N_2_O emissions. Moreover, at the end of the experiment no changes were observed in the ratio (*nosZI* + *nosZII*/*nirS* + *nirK*), which indicates that at high soil water content biochar addition did not promote the reduction of N_2_O to N_2_. Although, at first, this may seem to contradict previous findings, we need to bear in mind that bacterial denitrification is not necessarily the main N_2_O formation pathway at 80%WFPS. Soils are complex environments where ammonia oxidation and nitrifier denitrification generally coexist with heterotrophic denitrification^75^, and the quantity of N_2_O produced in each route depends not only on the WFPS, but also on different factors like soil N and organic C contents, O_2_ pressure or pH^76,77^. Our hypothesis is that biochar did not mitigate N_2_O emissions at high moisture content because its application did not affect nitrifier populations, and the changes in denitrifying communities were minimal, probably not enough to reflect changes in N_2_O emissions.

### DMPP and biochar: a negative synergy

A highly significant interaction was demonstrated between biochar amendment and fertilization affecting N_2_O emissions, soil ammonium and nitrate contents and ammonia oxidizing populations (Table 2). This interaction indicated that the reduction observed in the efficiency of DMPP mitigating N_2_O emissions in biochar amended soils was significant. To date, few studies have reported effects of biochar application on the efficiency of NIs. Treweek *et al*.^78^ reported no differences in N_2_O emissions when applying DCD alone or combined with biochar under field conditions. In a laboratory study, Shi *et al*.^79^ described lower DCD nitrification inhibitory ability when applied in combination with biochar. Given that DMPP reduces N_2_O emissions by inhibiting the microbial nitrification pathway, and if biochar amendment has been proposed as a potential tool to also mitigate N_2_O emissions, the addition of DMPP to biochar amended soils was expected to be a further benefit. However, results demonstrated a counteracting effect between biochar and DMPP: biochar diminished the efficiency of DMPP at both low and high soil water contents. These higher N_2_O emissions in biochar amended soils in relation to control soils when DMPP was applied were concomitant with greater nitrifier populations and higher nitrate contents. The decrease observed in AOB populations due to DMPP application at 80% WFPS was counteracted in the biochar amended soil (Fig. 3) even by the end of the second phase. Similarly, at 40% WFPS DMPP did not further reduce the yet lowered AOB population following biochar amendment (Fig. 3). This counteracting effect over AOB populations restored the competition between AOB and AOA at both soil water contents, leading to lower AOA/AOB ratios in biochar amended soils when DMPP was applied with respect to the non-amended (Fig. 5). By the end of the experiment, the induction of *nosZ*-bearing microorganisms observed at low soil water content when DMPP was applied respecting to AS application was also counteracted in the biochar amended soil (Fig. 2).

To explain this counteracting effect observed between biochar and DMPP, different mechanisms can be proposed. Electrostatic attraction, polar and non-polar organic-attraction and ion-exchange to the carbonized phase of biochar, and surface sorption to the non-carbonized phase are some of the mechanisms involved in the interactions of biochar with organic and inorganic contaminants^80^, reducing the efficiency of DMPP. One hypothesis to explain this reduction of the DMPP efficiency is that, due to its varied surface functional groups, micro-porous structure and large specific surface area, biochar has a strong affinity and adsorption ability for organic substances^79^. Thus, the ability of biochar to reduce the efficiency of DMPP may be explained by means of an adsorption process, as also described for the reduction in the bioavailability of heavy metals and/or pesticides in soil when biochar was added^81,82^. Attending to the DMPP chemical structure, which has a protonated N, it might be probable that DMPP is immobilized or adsorbed by the carboxyl groups of biochar by means of electrostatic attraction. Due to biochar hydrophobicity and the uncharged status of the DMPP molecule at neutral pH, hydrophobic interactions between biochar and DMPP have been hypothesized as a plausible reason for the sorption^27^. However, no data were reported about the availability of DMPP for microbes or its inhibitory effect on N_2_O emissions. To our knowledge, this is the first study reporting a negative synergy between this dimethylpyrazole-based nitrification inhibitor and a biochar. Further experiments are necessary to clarify the kinetics of this sorption, since whereas Keiblinger *et al*.^27^ described a DMPP sorption after 24 hours of shaking DMPP with soil solution, under our experimental conditions at least 30 days were necessary for biochar to decrease DMPP efficiency inhibiting nitrification and subsequent N_2_O emissions.

## Conclusions

This study demonstrates that DMPP significantly reduced the N_2_O emissions produced by the application of ammonium sulphate by 72–89% at both low and high soil water content conditions. This reduction was due to a dual effect decreasing the ammonia-oxidizing bacterial population on the one hand and inducing changes in the denitrifiers populations that can promote the reduction of N_2_O to N_2_ in the last step of denitrification on the other. The study also demonstrates that Loblolly pine biochar mitigates N_2_O emissions in soils at low soil water contents by reducing the abundance of nitrifying bacteria. However, the combined application of DMPP and biochar significantly reduced the nitrification inhibitory effect of DMPP and the subsequent mitigation of N_2_O emissions at both low and high soil water content conditions, probably due to the adsorption of DMPP to biochar surfaces. Further experiments are needed to understand the basis of this adsorption mechanism and its dynamics along the time in view of improving N_2_O emission mitigation strategies^83^.

## Supplementary information

Supplementary Table 1
