# Supplementary material for: Biochar reduces the efficiency of nitrification inhibitor 3,4-dimethylpyrazole phosphate (DMPP) mitigating N2O emissions
Source: Sci Rep. 2019 Feb 20;9:2346. doi: 10.1038/s41598-019-38697-2 (PMC6382844; doi:10.1038/s41598-019-38697-2)
Supplement: Supplementary file 1 — Supplementary Table 1 [file 41598_2019_38697_MOESM1_ESM.pdf]

Biochar reduces the efficiency of nitrification inhibitor 3,4-dimethylpyrazole phosphate (DMPP) mitigating N_2O emissions

Fuertes-Mendizábal T[1], Huérfano X[1], Vega-Mas I[1], Torralbo F[1], Menéndez S[1], Ippolito JA[2], Kammann C[3], Wrage-Mönnig N[4], Cayuela ML[5], Borchard N[6,7], Spokas K[8], Novak J[9], González-Moro MB[1], González-Murua C[1] and Estavillo JM[1]

Supplementary Table 1. Primers pairs and thermal conditions used for real-time qPCR.

Target group	Primer name	Sequence	Thermal profile	bp length	Efficiency (%)	References
16S rRNA Bacteria	341F<br>534R	5´-CCTACGGGAGGCAGCAG-3´<br>5´-ATTACCGCGGCTGCTGGCA-3´	95ºC for 2 min – x 1 cycle<br>95ºC for 15 sec , 60 °C for 30 sec , 72 °C for 30 sec , 80 °C for 30sec – x 40 cycles	174	95	Lopez-Gutiérrez et al., (2004)
16S rRNA Archaea	771F<br>957R	5´-ACGGTGAGGGATGAAAGCT-3´<br>5´ -CGGCGTTGACTCCAATTG-3´	95 °C for 2 min – x 1 cycle<br>95 °C for 15 sec , 58 °C for 30 sec , 72 °C for 30 sec , 80 °C for 30sec – x 40 cycles	226	93	Ochsenreiter et al., (2003)
Bacterial amoA	amoA1F<br>amoA2R	5´-GGGGTTTCTACTGGTGGT-3´<br>5´-CCCTCKGSAAAGCCTTCTTC-3´	95 °C for 2 min – x 1 cycle<br>95 °C for 15 sec, 54 °C for 60 sec , 72 °C for 60 sec – x 40 cycles	491	93	Rotthauwe et al., (1997)
Archaea amoA	Arch-amoAF<br>Arch-amoAR	5´-STAATGGTCTGGCTTAGACG-3´<br>5´-GCGGCCATCCATCTGTATGT-3´	95 °C for 2 min - x 1 cycle<br>95 °C for 45 sec, 54 °C for 45 sec, 72 °C for 45 sec; 85 °C for 20 sec - x 40 cycles	635	86	Francis et al., (2005)
narG	NarG-f<br>NarG-r	5'-TCGCCSATYCCGGCSATGTC-3'<br>5'-GAGTTGTACCAGTCRGCSGAYTCSG-3'	95 °C for 2 min – x 1 cycle<br>95 °C for 15 sec, 63 °C for 30 sec (-1 °C /cycle), 72 °C for 30 sec, 80 °C for 30 sec – x 6 cycles<br>95 °C for 15 sec, 58 °C for 30 sec, 72 °C for 30 sec, 80 °C for 30sec – x 40 cycles	173	98	Bru et al., (2007)
nirS	cd3aF<br>R3cd	5´-GTSAACGTSAAGGARACSGG-3´<br>5´-GASTTCGGRTGSGTCTTGA-3´	95 °C for 2 min - x 1 cycle<br>95 °C for 45 sec, 55 °C for 45 sec, 72 °C for 45 sec; 85 °C for 20 sec - x 40 cycles	410	86	Michotey et al., (2000)<br>Throbäck et al., (2004)
nirK	NirK 876<br>NirK1040	5´-ATYGGCGGVCAYGGCGA-3´<br>5´-GCCTCGATCAGRTTRTGGTT-3´	95 °C for 2 min – x 1 cycle<br>95 °C for 15 sec, 63 °C for 30 sec (-1 °C /cycle), 72 °C for 30 sec, 80 °C for 15 sec – x 6 cycles<br>95 °C for 15 sec, 58 °C for 30 sec, 72 °C for 30 sec, 80 °C for 30sec – x 40 cycles	165	93	Henry et al., (2004)
nosZI	nosZ-F<br>nosZ-R	5´-CGCRACGGCAASAAGGTSMSSGT-3´<br>5´-CAKRTGCAKSGCRTGGCAGAA-3´	95 °C for 2 min – x 1 cycle<br>95 °C for 15 sec, 65 °C for 30 sec (-1 °C /cycle), 72 °C for 30 sec, 80°C for 30 sec – x 6 cycles<br>95 °C for 15 sec, 60 °C for 30 sec, 72 °C for 30 sec, 80 °C for 30sec – x 40 cycles	267	90	Henry et al., (2006)
nosZII	nosZ-II-F<br>nosZ-II-R	5´-CTIGGICCIYTKCAYAC-3´<br>5´-GCIGARCARAAITCBGTRC-3´	95 °C for 2 min – x 1 cycle<br>95 °C for 30 sec, 54 °C for 30 sec, 72 °C for 40 sec, 85 °C for 15 sec - x 40 cycles	698	69	Jones et al., (2013)
